# MicroRNA-185 inhibits proliferation by targeting c-Met in human breast cancer cells

**DOI:** 10.3892/etm.2014.1999

**Published:** 2014-09-30

**Authors:** PEIFEN FU, FEIYA DU, MINYA YAO, KEZHEN LV, YU LIU

**Affiliations:** 1 Department of Breast Surgery, The First Affiliated Hospital, Zhejiang University School of Medicine, Hangzhou, Zhejiang 310003, P.R. China; 2 Department of Plastic Surgery, The First Affiliated Hospital, Zhejiang University School of Medicine, Hangzhou, Zhejiang 310003, P.R. China

**Keywords:** breast cancer, cell proliferation, miR-185, c-Met

## Abstract

MicroRNAs (miRNAs) are a group of small non-coding RNA molecules that have been shown to regulate the expression of genes involved in tumorigenesis. The relevance of miRNAs in the development, progression and prognosis of human breast cancer is not fully understood. miR-185 has been demonstrated to be involved in the pathogenesis of several types of cancers; however, its role in breast cancer has not yet been elucidated. In the present study, the expression of miR-185 was analyzed by quantitative polymerase chain reaction. In addition, an MTT assay and flow cytometry were used to determine the rates of cell proliferation and apoptosis. Protein expression was analyzed by western blotting and the target gene was confirmed using a luciferase reporter assay. The expression of miR-185 was found to be downregulated in the breast cancer tissues. The MTT assay revealed that overexpression of miR-185 inhibited the proliferation of MDF7 and SKBR3 cells. Furthermore, flow cytometric analysis demonstrated that increased expression levels of miR-185 promoted the apoptosis of breast cancer cells. In addition, c-Met expression was demonstrated to be significantly upregulated in breast cancer tissues and cells, and the c-Met gene was identified to be a target of miR-185. Therefore, the results demonstrated that miR-185 inhibited the proliferation of breast cancer cells by regulating the expression of c-Met, indicating its potential as a therapeutic target for breast cancer.

## Introduction

Breast cancer is the most frequently diagnosed cancer and the leading cause of mortality from cancer among females, accounting for 23% of total cancer cases and 14% of cancer mortalities (1,2). Despite the development of surgical techniques and meticulously designed chemotherapy regimens, relapse remains almost inevitable in patients with advanced cases of the disease. Although there are a number of chemical therapeutic drugs for the treatment of breast cancer that are able to kill or inhibit the growth of tumors, they are usually associated with a number of side-effects (3,4). Therefore, further investigation into the molecular pathogenesis of breast cancer and the identification of novel and effective biomarkers are urgently required.

MicroRNAs (miRNAs) are a class of small, non-coding RNAs, which are capable of regulating the expression of genes at the post-transcriptional level (5,6). Mechanistically, miRNA functions by binding to the 3′ untranslated region (UTR) of target mRNA, blocking translation and/or causing mRNA degradation (7). Previous investigations have demonstrated that miRNAs play a diverse role in tumorigenesis and may function as oncogenes, tumor suppressors and modulators of tumor proliferation, apoptosis and drug resistance (8–10). Among numerous miRNAs, miR-185 stands out as an important molecule. Analyses of ovarian cancer, pediatric renal tumor and prostate cancer cases have revealed a decreased expression of miR-185, which may be involved in tumor initiation and progression (11,12). However, the biological function and underlying molecular mechanisms of miR-185 in breast cancer have not been fully elucidated. Therefore, in the current study, the association between miR-185 and breast cancer was investigated.

## Materials and methods

### Cell culture and tissue samples

Two human breast cancer cell lines (MCF7 and SKBR3) and a normal human mammary epithelial cell line (MCF10A) were obtained from the Chinese Academy of Sciences (Shanghai, China). All the cell lines used were cultured in RPMI 1640 medium (Gibco Life Technologies, Beijing, China) supplemented with 10% fetal calf serum, 100 IU/ml penicillin and 100 mg/ml streptomycin (Gibco Life Technologies). In addition, human breast cancer tissues and distant normal tissues were collected during routine therapeutic surgery at the Department of Breast Surgery, the First Affiliated Hospital of Zhejiang University School of Medicine (Hangzhou, China). Written informed consent was obtained from all participants involved in this study. The study was performed in accordance with the Declaration of Helsinki and was approved by the Institutional Review Board of Zhejiang University (Hangzhou, China).

### RNA extraction and quantitative analysis

Cells were seeded into 12-well plates and total RNA was isolated using TRIzol reagent (Invitrogen Life Technologies, Carlsbad, CA, USA), according to the manufacturer’s instructions. RNA was reverse transcribed and amplified using a real time-polymerase chain reaction (PCR) miRNA detection kit (Ambion Life Technologies, Carlsbad, CA, USA), according to the manufacturer’s instructions. PCR was performed using an ABI 7500 Real-Time PCR System (Applied Biosystems, Carlsbad, CA, USA) with the following conditions: One cycle of 95°C for 10 min; and 40 cycles of 95°C for 15 sec and 60°C for 1 min. The U6 small nuclear RNA was used as the control. The mRNA expression of c-Met was measured by quantitative PCR (qPCR), with GAPDH used as the control. The primer sequences were as follows: Forward, 5′-CAGATGTGTGGTCCTTTG-3′, and reverse, 5′-ATTCGGGTTGTAGGAGTCT-3′.

### MTT assay

Cell proliferation was determined using an MTT assay. The cells were seeded into 96-well plates at a density of 3×10^4^ cells/well. Next, 10 ml MTT (5 mg/ml; Sigma-Aldrich, St. Louis, MO, USA) was added and the plates were incubated in the dark at 37°C for 2 h. The absorbance was determined using a Model 680 Microplate Reader (Bio-Rad Laboratories, Inc., Hercules, CA, USA) at a wavelength of 490 nm.

### Apoptosis analysis

At 48 h post-transfection with the miR-185 mimics/inhibitor or control, the cells were washed with phosphate-buffered saline (PBS), detached with trypsin and harvested. Subsequently, the cells (1×10^6^) were centrifuged at 700 × g for 5 min and the supernatant solutions were discarded. The cells were washed twice with PBS, 70% alcohol was added and the mixture was centrifuged at 700 × g for 5 min. Apoptotic cells were evaluated using the Annexin V-FITC/PI Cell Apoptosis Detection kit (BD Pharmingen, San Diego, CA, USA), following the manufacturer’s instructions.

### Western blotting

Cultured cells were lysed using radioimmunoprecipitation assay buffer, and tissue samples were lysed using T-PER Tissue Protein Extraction Reagent (Sigma) in the presence of a protease inhibitor cocktail (Pierce Biotechnology, Inc., Rockford, IL, USA). The tissue and cell lysates were subjected to sodium dodecyl sulfate polyacrylamide gel electrophoresis. For immunoblotting, the membranes were blocked with 5% non-fat milk in Tris-buffered saline, and incubated with a mouse anti-human c-Met monoclonal antibody (Abcam, Cambridge, MA, USA), followed by a horseradish peroxidase-conjugated secondary antibody (Abcam). The signals were detected using Immobilon (Millipore, Billerica, MA, USA) and the immunoreactive bands were identified using an enhanced chemiluminescence kit (Sigma) for western blotting detection and a ChemiGenius bioimaging system (Syngene, Frederick, MD, USA). GAPDH levels were measured as a loading control.

### Plasmid construction and luciferase activity assay

In order to perform the fluorescent reporter assay, the following primers were used to amplify the 3′-UTR of the c-Met gene: Forward, 5′-GATCCTGCTAGTACTATGTCAAAGCAACAGTC-3′, and reverse, 5′-AATTCTCAGGCAGTGAAAAAACCATTGGAC-3′. Subsequently, a plasmid containing the 3′-UTR of c-Met and a fluorescent reporter was constructed. MCF7 cells were seeded into 48-well plates and cotransfected with mimic control, miR-185 mimics or miR-185 inhibitor. The enhanced green fluorescent protein (EGFP) activity was normalized against the red fluorescent protein activity. After 72 h, the fluorescence intensity was determined using a fluorescence spectrophotometer (Hitachi, Ltd., Tokyo, Japan). The primers were designed by Primer Premier 5.0 (Premier Biosoft, Palo Alto, CA, USA).

### Statistical analysis

All the data are presented as the mean ± standard error of mean and were analyzed using SPSS 13.0 software (SPSS, Inc., Chicago, IL, USA). For comparisons between two groups, the statistical significance was determined using the Student’s t-test. Comparisons among groups were performed using analysis of variance. P<0.05 was considered to indicate a statistically significant difference.

## Results

### miR-185 is downregulated in breast cancer tissue

**Figure 1:**
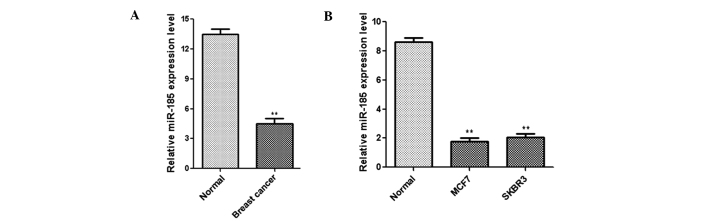
Downregulation of miR-185 in human breast cancer tissues. (A) Expression levels of miR-185 were determined in human breast cancer and adjacent non-tumor tissues by quantitative polymerase chain reaction (qPCR). (B) A normal human mammary epithelial cell line (MCF10A) and two breast cancer cell lines (MCF7 and SKBR3) were analyzed by qPCR to measure the expression level of miR-185. ^**^P<0.01, vs. normal.

To investigate the clinical relevance of miR-185 in human breast cancer, miR-185 expression was analyzed in 24 paired breast cancer and adjacent non-tumor tissues. qPCR analysis indicated that the expression level of miR-185 was clearly downregulated in the cancer tissues when compared with the corresponding non-tumor samples (Fig. 1A). In addition, the normal human mammary epithelial cell line (MCF10A) and breast cancer cell lines (MCF7 and SKBR3) were analyzed with qPCR. A significant downregulation in the expression level of miR-185 was observed in the breast cancer cell lines when compared with the normal cell line (Fig. 1B). These results indicated that expression levels of miR-185 are decreased significantly in breast cancer tissues and cell lines.

### Effects of miR-185 on breast cancer cell proliferation

**Figure 2:**
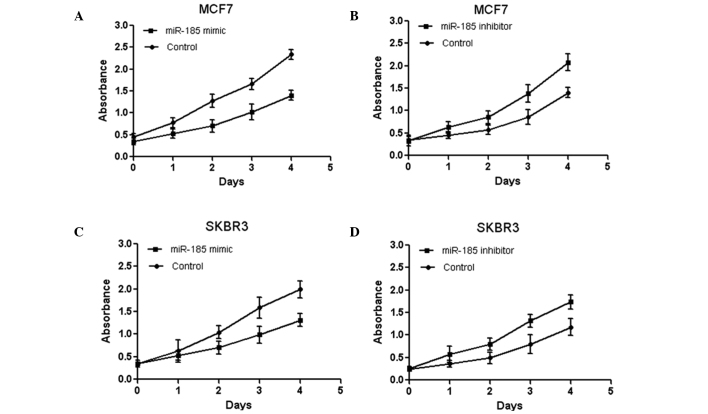
Effects of miR-185 on breast cancer cell proliferation. The MDF7 human breast cancer cell line was transfected with (A) miR-185 mimics or (B) inhibitor, and the SKBR3 breast cancer cell line was transfected with (C) miR-185 mimics or (D) inhibitor. An MTT assay was performed every 24 h for the determination of cell proliferation.

To investigate the effect of miR-185 on breast cancer cell proliferation, miR-185 mimics was transfected into the human breast cancer cell lines, MCF7 and SKBR3, and the proliferation was assessed by an MTT assay. The data indicated that overexpression of miR-185 significantly inhibited MCF7 cell proliferation (Fig. 2A). In addition, the MCF7 cells were transfected with an miR-185 inhibitor and were found to exhibit increased proliferation, as demonstrated by an MTT assay (Fig. 2B). Similar results were observed for the SKBR3 cells (Fig. 2C and D). Collectively, these results demonstrated that miR-185 inhibited breast cancer cell proliferation *in vitro*.

### Overexpression of miR-185 promotes breast cancer cell apoptosis

**Figure 3:**
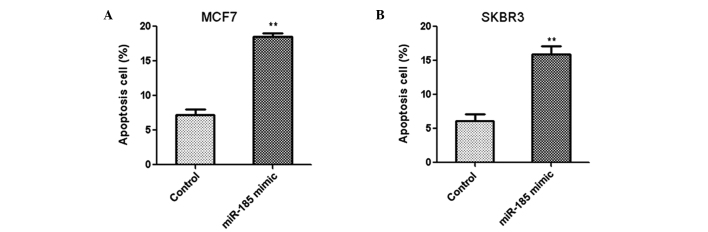
Overexpression of miR-185 promotes the apoptosis of breast cancer cells. The effect of miR-185 on the apoptosis of human breast cancer cells was investigated by flow cytometry. Apoptosis rates were determined in (A) MCF7 and (B) SKBR3 breast cancer cells lines following transfection with miR-185 mimics. ^**^P<0.01, vs. control.

The effect of miR-185 on the apoptosis of human breast cancer cells was investigated using flow cytometry. Two breast cancer cells lines, MCF7 and SKBR3, were transfected with miR-185 mimics and the apoptosis rate was analyzed using annexin V/propidium iodide staining. The results indicated that overexpression of miR-185 led to a significant increase in the apoptosis rates of MCF7 (Fig. 3A) and SKBR3 (Fig. 3B) cells.

### c-Met is a target of miR-185 in breast cancer cells

**Figure 4:**
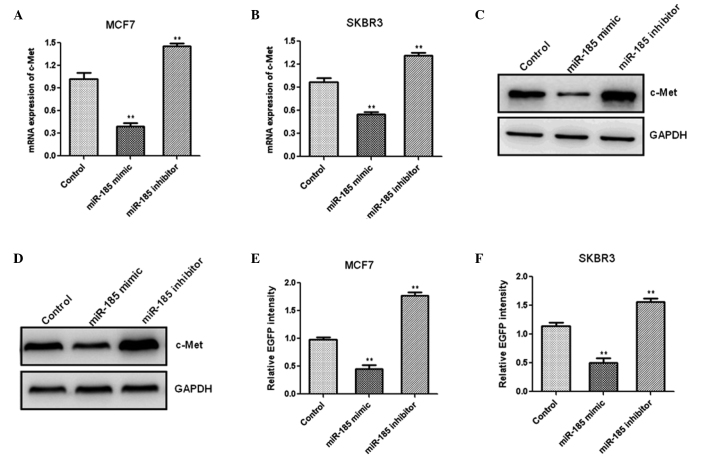
c-Met was found to be a target of miR-185 in breast cancer cells. The effect of miR-185 on the mRNA expression of c-Met in (A) MCF7 and (B) SKBR3 cells, and on the protein expression of c-Met in (C) MCF7 and (D) SKBR3 cells. The miR-185 binding sites in the 3′ untranslated region of c-Met were assessed using fluorescent reporter assays in (E) MCF7 and (F) SKBR3 cells. Cells were transfected with a control, miR-185 mimics or miR-185 inhibitor. GAPDH was used as an internal control. ^**^P<0.01, vs. control. EGFP, enhanced green fluorescent protein.

To investigate the mechanism by which miR-185 inhibits cell proliferation in breast cancer tissues, putative miR-185 targets were analyzed using the miRanda, TargetScan and PicTar software. The 3′-UTR of c-Met, containing the putative miR-185 binding sites, was identified. To determine whether miR-185 targeted c-Met *in vitro*, the MCF7 cells were transfected with miR-185 mimics or an inhibitor, and the mRNA expression of c-Met was detected using qPCR. Overexpression of miR-185 significantly decreased the mRNA expression of c-Met when compared with the controls, whereas inhibition of miR-185 resulted in an increase in c-Met mRNA expression in MCF7 (Fig. 4A) and SKBR3 (Fig. 4B) cells. Western blotting demonstrated that overexpression of miR-185 resulted in an evident decrease in c-Met protein expression, while a reduction in miR-185 markedly increased the protein expression of c-Met in the MCF7 (Fig. 4C) and SKBR3 cells (Fig. 4D). These results indicated that miR-185 regulated the mRNA and protein expression levels of c-Met. Fluorescent reporter assays were performed to determine whether c-Met was a direct target of miR-185. The 3′-UTR of c-Met with the predicted binding site for miR-185 was cloned into a fluorescent reporter vector. Upregulation of miR-185 expression reduced the intensity of EGFP in the cells transfected with a vector containing the c-Met 3′-UTR when compared with the control groups, whereas in the miR-185 inhibitor group, the intensity of EGFP in the MCF7 and SKBR3 cells increased significantly (Fig. 4E and F). These results indicated that miR-185 binds to the 3′-UTR of c-Met directly.

## Discussion

miRNAs are a group of small non-coding RNAs that modulate the expression of genes by targeting mRNAs for translational repression (13). Thus, the key to understanding the function of miRNA is the elucidation of functional targets, which usually involves analyzing changes in the target proteins following a gain or loss of function in the specific miRNA. In the present study, for the first time, miR-185 was demonstrated to inhibit the proliferation of breast cancer cells by regulating the expression of c-Met.

Aberrant expression of miRNAs plays a critical role in cell proliferation, apoptosis and cell cycle arrest in various cancer types (14,15). A recent study demonstrated that miR-155 promoted the proliferation of human breast cancer MCF-7 cells through targeting tumor protein 53-induced nuclear protein 1; thus, provided a new therapeutic strategy for breast cancer (16). An additional study revealed that miR-24 regulated cell proliferation and DNA repair directly. The study hypothesized that enhancing miR-24 function in cancer cells by introducing miR-24 mimics may be an attractive therapeutic method, as miR-24 may potentially block dysregulated cell proliferation and sensitize cancer cells to DNA damage from chemotherapy and radiotherapy (17). In the present study, the MTT assay revealed that overexpression of miR-185 significantly inhibited the proliferation of MCF7 and SKBR3 cells. In addition, human breast cancer cells transfected with an miR-185 inhibitor exhibited increased proliferation, indicating that miR-185 inhibited the proliferation of breast cancer cells *in vitro*.

Apoptosis is the process of programed cell death, which is associated with cell growth and maintaining cellular homeostasis (18). Increasing evidence has shown that miRNAs play a critical role in cell proliferation, differentiation and apoptosis (14). A recent study revealed that downregulation of miR-155 induced cell apoptosis by targeting a number of antiapoptotic factors and causing cell cycle arrest (19). Furthermore, a previous study demonstrated that miR-185 targeted the expression of RhoA and Cdc42, and inhibited the proliferation potential of human colorectal cells (20). The results of the present study demonstrated that overexpression of miR-185 promoted the apoptosis of MCF7 and SKBR3 cells.

miRNAs control cellular biological functions by targeting the expression of genes; therefore, the elucidation of functional targeted genes is crucial. Studies on signal transduction pathways have generated various promising molecular targets for therapeutic inhibition in cancer therapy (13). Receptor tyrosine kinases represent an important class of such therapeutic targets. c-Met is a receptor tyrosine kinase that has been shown to be overexpressed in a variety of malignancies (21,22). Increased c-Met signaling promotes cell migration and invasion through several pathways, including the extracellular signal-regulated kinase, phosphatidyl inositol 3-kinase and focal adhesion kinase pathways (23,24). Consistent with observations of previous studies, the present study demonstrated that the expression level of c-Met increased significantly in breast cancer samples. In addition, transfection with miR-185 mimics resulted in decreased luciferase activity and c-Met expression in breast cancer cells, indicating that c-Met is the target gene of miR-185.

In conclusion, miR-185 was found to be significantly downregulated in breast cancer tissues. Moreover, miR-185 was demonstrated to inhibit the proliferation of breast cancer cells by regulating the expression of c-Met, which indicates the therapeutic potential of miR-185 in breast cancer treatment.
